# Demonstrating a Quartz Crystal Microbalance with Dissipation (QCMD) to Enhance the Monitoring and Mechanistic Understanding of Iron Carbonate Crystalline Films

**DOI:** 10.1021/acs.langmuir.3c03150

**Published:** 2024-07-09

**Authors:** Igor Efimov, Eftychios Hadjittofis, Mustafa M. Alsalem, Kyra L. Sedransk Campbell

**Affiliations:** † Department of Chemical and Biological Engineering, University of Sheffield, Mappin Street, Sheffield S1 3DJ, U.K.; § Department of Chemical Engineering, Imperial College London, South Kensington Campus, London SW7 2AZ, U.K.

## Abstract

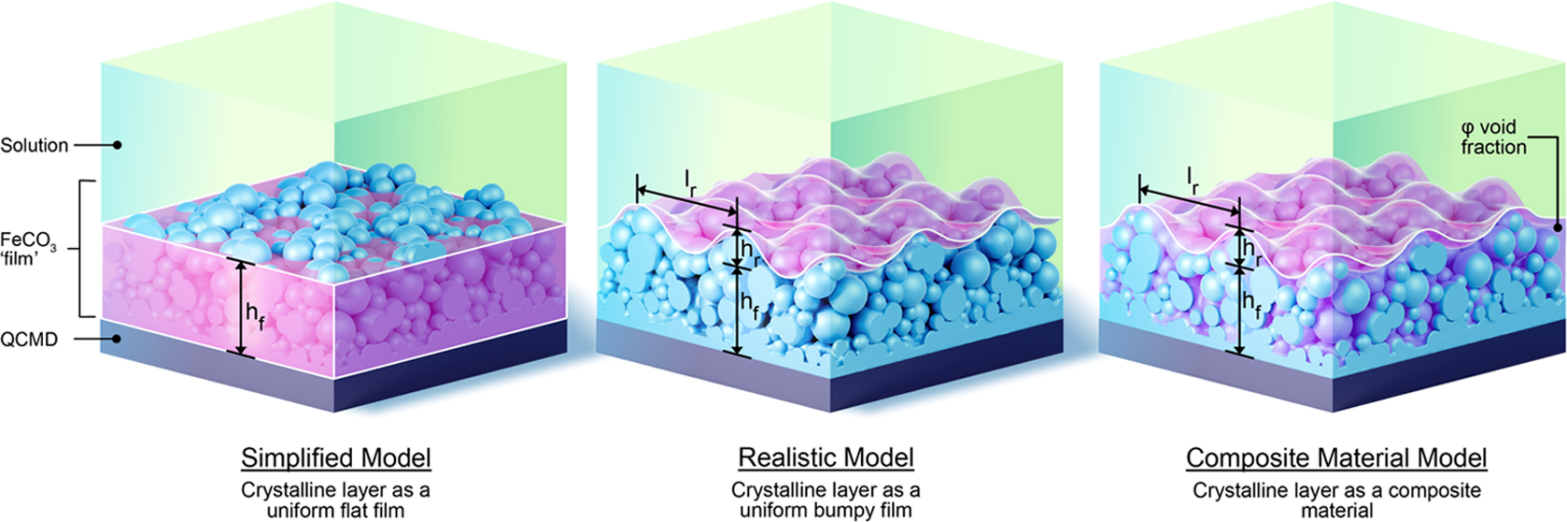

This paper reports the real time monitoring of siderite deposition, on both Au- and Fe-coated surfaces, using the changes in frequency and dissipation of quartz crystal microbalance with dissipation (QCMD). In an iron chloride solution saturated with carbon dioxide, buffered with sodium bicarbonate to pH 6.8, roughly spherical particles of siderite formed within 15 min, which subsequently deposited on the QCMD crystal surface. Imaging of the surface showed a layer formed from particles ca. < 0.5 μm in diameter. Larger particles are clearly deposited on top of the lower layer; these larger particles are >1 μm in diameter. Monitoring of the frequency clearly differentiates the formation of the lower layer from the larger crystals deposited on top at later times. The elastic moduli calculated from QCMD data showed a progressive dissipation increase; the modeling of the solid–liquid interface using a flat approximation resulted in a poor estimation of elastic and storage moduli. Rather, the impedance modeled as a viscoelastic layer in contact with a semi-infinite liquid, where a random bumpy surface with a Gaussian correlator is used, is much more accurate in determining the elastic and storage moduli as losses from the uneven interface are considered. A further step considers that the film is in fact a composite consisting of hard spherical particles of siderite with water in the vacant spaces. This is treated by considering the individual contributions of the phases to the losses measured, thereby further improving the accuracy of the description of the film and the QCMD data. Collectively, this work presents a new framework for the use of QCMD, paired with traditional approaches, to enhance the understanding of crystal deposition and film formation as well as quantify the often evolving mechanical properties.

## Introduction

A wide range of iron-based infrastructure, primarily in the oil and gas industry, handles liquid flows containing large quantities of CO_2_. In the absence of oxygen, Fe^2+^ dissolved in the solution has the potential to form siderite (FeCO_3_). The formation of this siderite is favored by elevated temperatures (>50 °C) and pHs (>7).^1^ Depending on its properties, the siderite formed can impact the lifetime of the infrastructure, either positively or negatively. In particular, it can act as either a corrosion inhibitor or a promoter.

Slow growth on the surface of the infrastructure, regulated by slow iron diffusion, leads to the formation of a relatively dense layer of crystalline siderite; this can act as a corrosion inhibitor. Alternatively, when there is a strong driving force for the formation of siderite, this can occur in the bulk of the liquid. In this case, it should be possible to observe roughly spherical aggregates of microparticles that are not always fully crystalline. The fate of these particles will be determined from the infrastructure-specific conditions, but they are not expected to facilitate any corrosion inhibition.

In this paper, the bulk formation of siderite in carbon dioxide-saturated, oxygen-free solutions, at different temperatures, is established. It is demonstrated that at the same [Fe^2+^] the induction time increases with increasing temperature due to a decrease in the supersaturation of the solution with respect to siderite.

Then, the deposition behavior of siderite, formed in bulk, on both the inert gold (Au) surface of a QCMD sensor and the Fe-coated surface of a QCMD sensor is investigated. The measurements allow for the calculation of the mechanical properties of the deposited film. The results show good agreement of the calculated elastic modulus with values obtained independently, by experimental and computational means. The QCMD results and the subsequent calculations are used to demonstrate the concept of liquid entrapment below the deposited film, as well as to calculate the evolution of the population of particles deposited on the film. In this way, they are demonstrating the capability of the instrument to probe phenomena at the submicron level.

Overall, this work combines the accuracy of the QCMD with some established concepts in physical chemistry to demonstrate the potential of studying the evolution of siderite deposition on different types of surfaces, under different conditions, corresponding to different types of infrastructure. We consider this as the first step toward the development of a systematic framework for the study of reactive film formation and growth, which could be used for the design of process equipment and beyond.

### Quartz Crystal Microbalance with Dissipation for Films with Uneven (Bumpy) Surfaces

The primary tool employed herein is a quartz crystal microbalance with dissipation, QCMD,^2^ with support from traditional surface techniques used in the assessment and analysis of crystals (SEM, EDS, XRD). The advantage of using QCMD to study crystallization is both technique sensitivity and in situ monitoring capabilities, without compromising on a controlled environment. Previous efforts to study portions of a crystalline lifecycle using QCM (EQCM, QCMD) have been exceptionally limited.^3−5^ These studies collectively demonstrate the proof-of-concept capability of this technique to become an important method for studying crystallization phenomena. Herein, we build upon this work to demonstrate that both the frequency and dissipation changes can be utilized to provide insights into not only individual crystals but also the particle population development in a film-like layer on a solid surface. Moreover, with the development of a model derived from fundamental principles, the changing physical nature of the composition can be quantified.

The siderite layer is neither rigid nor viscous; it is neither infinitely large nor vanishingly thin. Furthermore, because the layer is composed of precipitated crystalline particles, it lacks uniformity. Therefore, existing models are inappropriate; the development of a new model must begin with the full equation for the acoustic impedance of a piezo resonator covered by a viscoelastic film, *Z*, which includes two acoustic impedance terms: for the film, *Z*_P_, (eq 1) and for the liquid, *Z*_L_, (eq 2). The acoustic impedance of the film *Z*_P_ is a function of film density ρ*_f_* and the complex shear modulus *G*
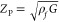
1The latter, *G*, itself is a sum of real and imaginary components: the storage modulus (*G*′*)* and loss modulus (*G*″), respectively (i.e., *G = G*′ *+ iG*″). The physical system includes a semi-infinite solution in contact with a viscoelastic film. We account for the acoustic impedance of this component
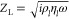
2by representing it as a shear wave propagating into the bulk. The parameters in the solution impedance are dynamic viscosity, η*_l_*, density, ρ*_l_*, and the shear acoustic wave function, ω *= 2*π*f*.^6,7^ The source of the oscillation is the solid surface of the piezoelectric quartz crystal. At this stage, the assumption remains that the viscoelastic layer is flat.
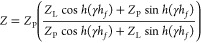
3In the definition of *Z* (eq 3), it is a function of *Z*_L_ (eq 2), *Z*_P_ (eq 1), γ, and *h*_*f*_; γ is the complex wavenumber of the shear acoustic wave, itself defined as γ = *i*ω*(*ρ_*f*_/*G)*^1/2^. This basis is well-known as a flawed simplification of the complexity of the siderite film layer; the layer itself is not flat and therefore cannot be approximated as such. As the film is formed by particle deposition it exhibits irregular/non-uniform porosity. Further, this porosity may be filled from the bulk liquid solution. The former issue, which could be described as roughness, will be introduced to correct the expression for *Z* (eq 3). The latter issue, porosity, does not require changes to the expression for *Z* (eq 3) explicitly; rather, it is in the extended interpretation of *G* values as effective values with contributions of both pores and crystals.

**Figure 1:**
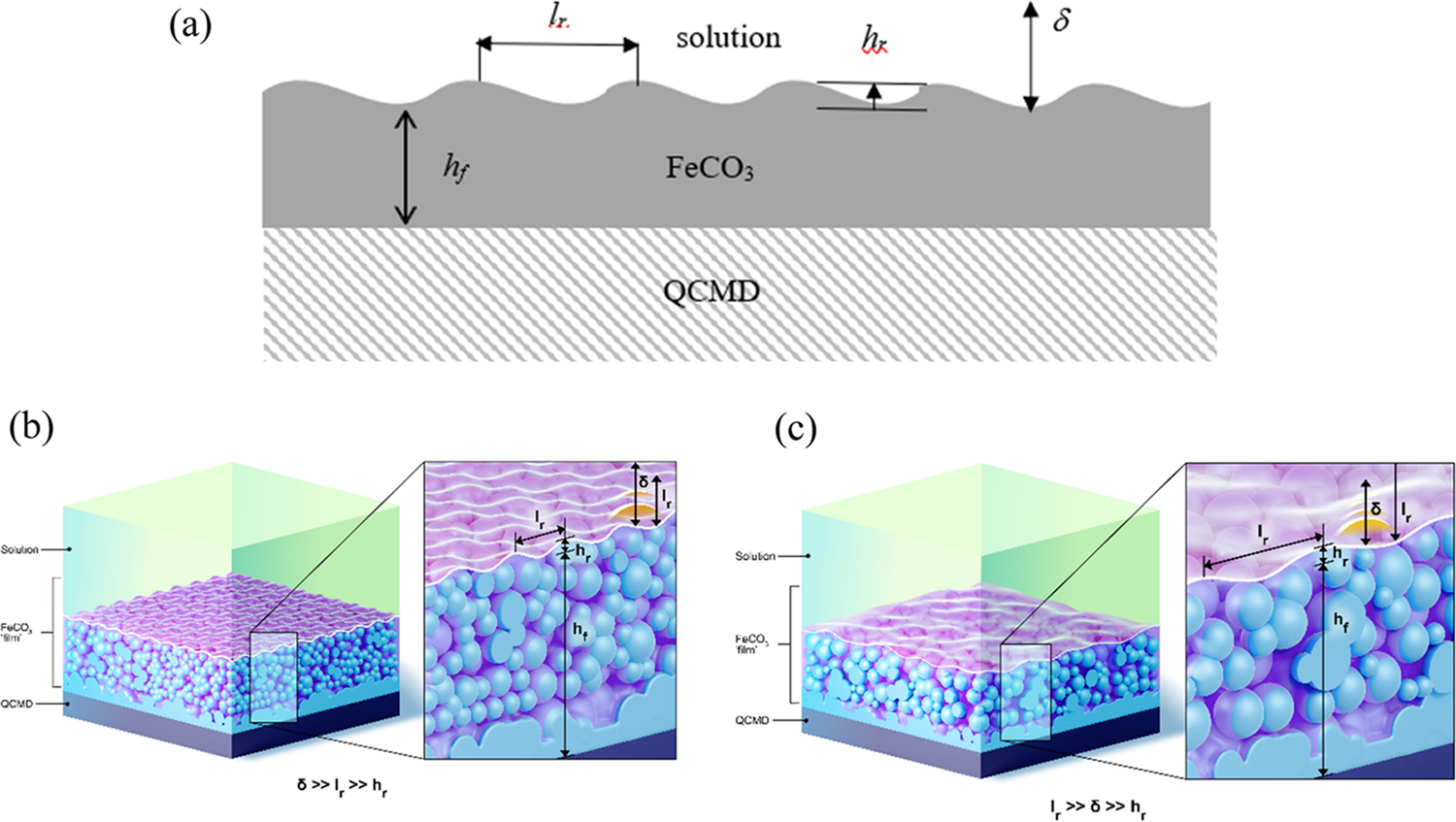
(a) 2D cartoon showing the QCM surface with a deposit having an uneven interface (bumpy) with the solution. 3-D renderings showing the two different cases: (b) as shown in eq 5 δ ≫ *l*_r_ ≫ *h*_r_ and (c) as shown in eq 6*l*_r_ ≫ δ ≫ *h*_r_.

As stated above, crystallization investigated in the current work does not produce flat interfaces; rather, these layers can be mathematically described as bumpy (corrugated is sometimes used in the literature) surfaces (Figure 1). Previously, *Z*_L_ for such interfaces had been calculated in^8^ for small roughness, δ; when *l*_r_ ≫ *h*_r_. The acoustic decay length (δ = (2η_*l*_/ρ_*l*_ω)^1/2^), correlation length along the surface (*l*_r_), and amplitude of the Gaussian correlator for random roughness (*h*) are parameters required for the 2D Fourier transform of deviation of height of the surface from an average value ξ(***K***) (eq 4). For this, ***K*** = (*K*_*x*_, *K*_*y*_) is the vector along the surface with random roughness, ξ(***K***), and *S* is the total area.
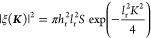
4

The two limiting cases allow explicit equations δ ≫ *l*_r_ in eq 5 and δ ≪ *l*_r_ in eq 6.^6−8^
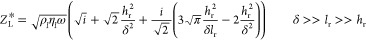
5
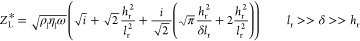
6
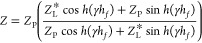
7

The first term in both cases is the Kanazawa–Gordon impedance for a flat interface (eq 2). To account for roughness, *Z*_L_^*^ is used, where it can take two possible forms, both modifications on eq 2: eq 5 or 6. The appropriate form is then employed in eq 7, akin to eq 2 used in eq 3. In ref (8), the asymptotic expansions eqs 5 and 6 were numerically compared with the exact solution in quadratures. It was found that in the region δ ∼ *l*_r_, all three methods result in differences of <10%. Therefore, this allows using the explicit expression of eq 5 or 6 for finding parameters of roughness in this region.

With the inclusion of an impedance term for film roughness, *G*′ can be calculated as a function of the various input parameters; appropriate selections of input parameters result in calculated *G*′ values, which approach both literature and computational values for siderite. Then, parameters of the precipitate film describe its roughness in Gaussian approximation, from which one can conclude about the size and spread of crystallites. Implementation of this approach cannot be achieved, yet, because of complexity of the problem. Therefore, certain assumptions were made, which will be described below.

## Experimental Section

Ferrous iron chloride hydrate FeCl_2_·4H_2_O (Aldrich, 99%) was dissolved in double-distilled water to the concentration of 5–25 mM. The solution was deaerated by nitrogen for 1 h and saturated by CO_2_ for 2 h. The solution pH was adjusted by adding sodium bicarbonate (NaHCO_3_) to reach a target pH of 6.8. Before using the solution, a few micrograms of sodium dithionate (Na_2_S_2_O_4_) were added. This was employed to reduce any remaining oxidized iron in ferric form as well as to reduce all remaining oxygen in solution (not removed by deaeration). Excess dithionite was employed to ensure that any diffusing oxygen was reduced; this kept the solution oxygen-free for hours, even in an open flask. A separate preliminary investigation demonstrated that as iron sulfates are all soluble, the addition of dithionite had no influence on crystallization.

5 MHz quartzes sputtered in-house with Au or Fe (AWSensors) were used with an open window cell seated in a high temperature-controlled chamber (Biolin QHTC101–0058) for a quartz crystal microbalance with dissipation (QCMD) (Biolin/QSense). The Au sensor was used several times. It was cleaned of previous deposits by 30% HCl or isopropyl alcohol or acetone. The Fe sensor was used only once as supplied, according to instructions of the manufacturer. A 0.6 mL droplet was transferred to the sensor surface. For isothermal experiments, the quartz was maintained at *T* = 80 °C with 0.1 °C accuracy; for nonisothermal experiments, a temperature ramp from 20 to 50 °C over 20 min was employed. The isothermal mode was used with Au-coated sensors, while the temperature ramp was used with Fe-coated sensors due to the instability of the Fe layer during prolonged exposure to elevated temperatures.

A 5 MHz fundamental frequency quartz plate was exposed to a solution in these experiments; the other electrode, exposed to air, was gold-coated. Several resonances are measured simultaneously at *nf* = 5, 15, 25... MHz, for *n* = 1, 3, 5... harmonic, respectively; the decay time of a pulse at each harmonic, which is called dissipation, *D*, was also recorded. The shift in resonance for a rigid deposit was used to ascertain the mass change and the dissipation of acoustic resistance.

Deposits on the crystal were analyzed ex situ by SEM (JeoL 6010), which included elemental analysis as well as XRD (Bruker D2).

## Results

### Siderite Crystallization in the Bulk of Solution

**Figure 2:**
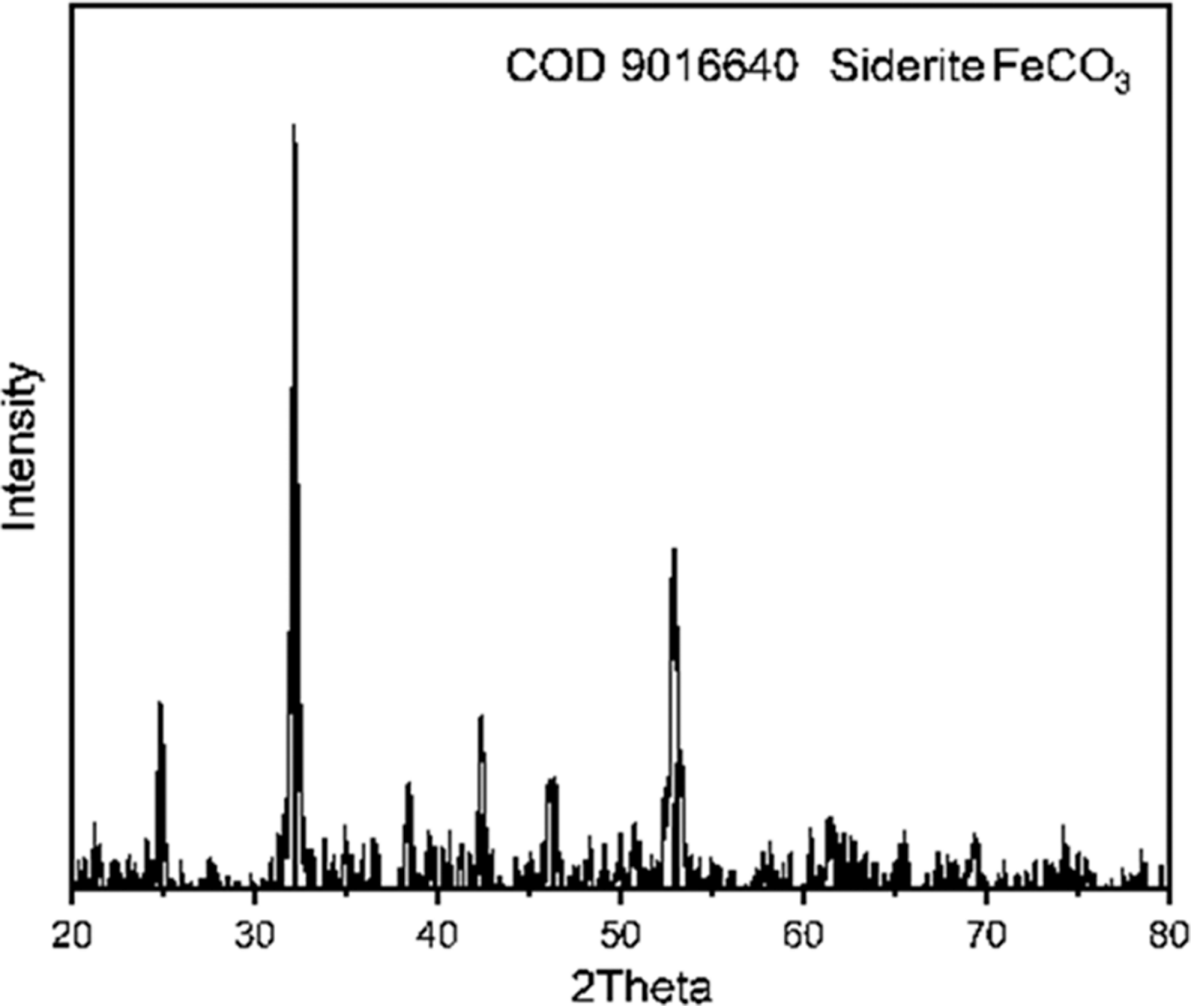
XRD spectrum of a siderite powder (COD 9016640) obtained in the bulk of 15 mM FeCl_2_, pH 6.8 adjusted with NaHCO_3_, deaerated and saturated with carbon dioxide. Precipitation began at 60 °C.

In an oxygen-free and carbon dioxide-saturated Fe^2+^ solution, as described in the Methods section, the turbidity of the solution was observed at various temperatures: 35 °C for 35 mM, 45 °C for 20 mM, 50 °C for 10 mM, and 60 °C for 5 mM ferrous iron. (These are reported for the qualitative value.) White crystals precipitated in all cases; at 90 °C, the solutions became visibly clear, with all apparent solid material at the bottom (white). The color of the precipitate remained white for 1 week when sodium dithionite was added to the liquid to keep it oxygen-free. Upon drying in air, the powder became brown in color; an XRD analysis of this solid indicates pure siderite (FeCO_3_) (Figure 2).

### Deposition on Au-Coated QCMD

**Figure 3:**
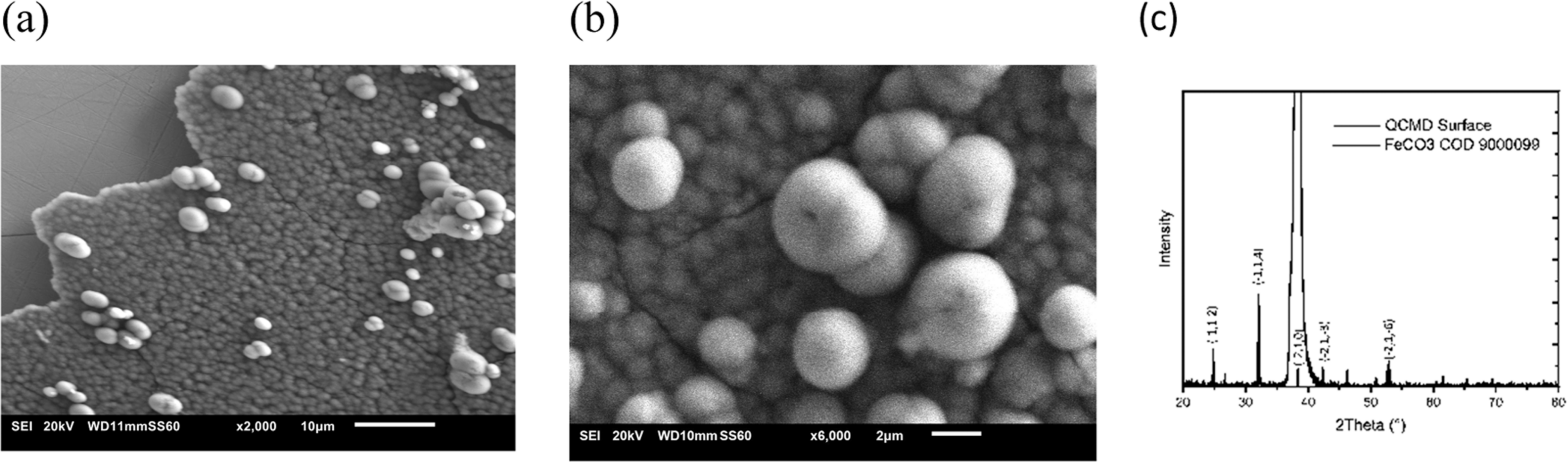
(a) SEM image of the dried siderite layer on Au-coated quartz. The film peeled off in a corner, which allows an estimate of its thickness. (b) Enlarged SEM image of (a) showing the location of siderite crystals of different sizes. (c) XRD spectrum of siderite deposits on Au-coated quartz. 15 mM FeCl_2_, pH 6.8 with NaHCO_3_, deaerated and saturated with carbon dioxide at 80 °C.

The presence of FeCO_3_ crystals on the Au-coated sensor can be confirmed by imaging analysis (SEM) (Figure 3a,b) as well as by clear peak matches as observed by XRD (Figure 3c), where Au and a single plane of SiO_2_ are the most prominent, but siderite is also clearly observed. Electron microscopy shows round, nearly spherical crystals (Figure 3b). Imaging shows an apparently more densely packed lower layer, with an estimated thickness of ca. 0.5 μm, consisting of <0.5 μm diameter particles. On the upper layer, imaging indicates that the particles are larger than those in the lower layer.

**Figure 4:**
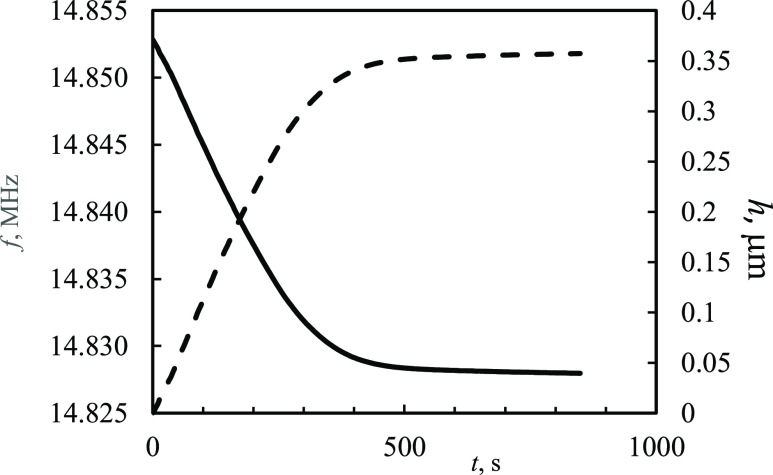
QCMD third harmonics frequency shift (*f*, solid line) and siderite film thickness growth (*h*, dashed line) estimated from the Sauerbray equation as a function of time. 15 mM FeCl_2_, pH 6.8 (with NaHCO_3_), *T* = 80 °C.

The formation of these crystalline layers was observed in situ; the data were recorded for the third resonance frequency (15 MHz), where the case study of 15 mM FeCl_2_ at 80 °C has been reported with the acoustic resistance as a function of time. The frequency drop (Figure 4), from *t* = 0 to *t* ≈ 400 s, is ca. 24 kHz. There are two clearly defined regions resulting from the frequency results: *t* < 400 s and *t* > 400 s. The former region shows an increase in mass on the surface, and the latter region shows virtually no change in mass. Assuming a siderite density of 3.96 g cm^–3^^1^ and 5 MHz QCM sensitivity 17.7 ng cm^–2^ Hz^–1^, the total frequency drop can be correlated with the formation of a layer that is 0.36 μm thick (=24 kHz × 17.7 ng/Hz cm^2^/3/3.96 g cm^–3^). This is the same order of magnitude as was observed from imaging obtained using SEM (Figure 4). The issue of uniformity and roughness will be considered with respect to dissipation.

**Figure 5:**
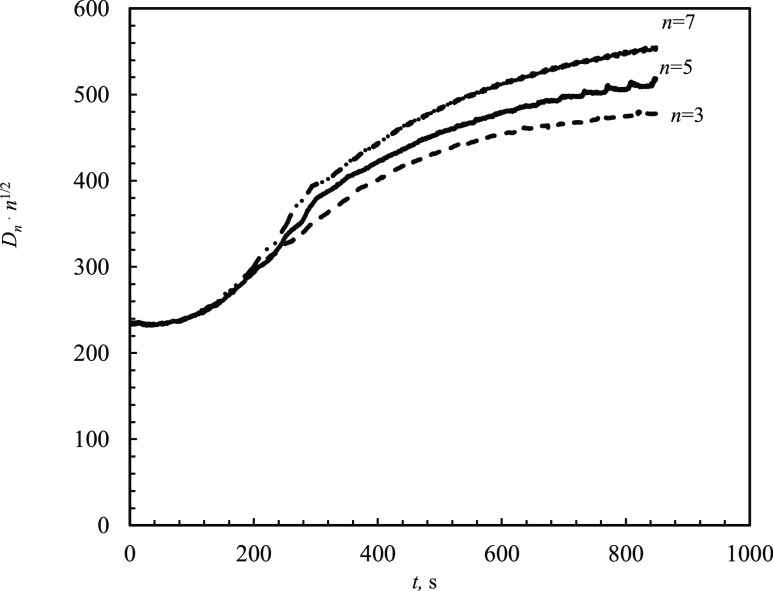
QCMD dissipation multiplied by *n*^1/2^ at different harmonics *n* = 3, 5, and 7 as a function of time during siderite precipitation from 15 mM FeCl_2_, pH 6.8 (with Na_2_CO_3_), *T* = 80 °C.

Although the frequency change was complete at ca. 400 s, the dissipation kept growing beyond this time, albeit at a smaller rate (Figure 5). Dissipation, *D*_*n*_, for three frequencies, *n* = 3, 5, 7, plotted as values *D*_*n*_·*n*^1/2^ as a function of time indicates a sequence of two different behaviors. Inspection of the behaviors indicates that at short times all three curves of *D*_*n*_·*n*^1/2^ coincide; beyond this initial time period, increasing differences among the frequencies are observed. For the case where there is a purely elastic load, *D*_*n*_ should coincide for all *n* and should not change. For the case where there is a purely viscous load with semi-infinite viscous media, *D*_*n*_·*n*^1/2^ should coincide for all *n* and should not change. (As a note, , where the acoustic resistance, *R*, is calculated as *R* = 2π*nfLD* with *L* = 35 mH for 5 MHz quartz.^6^) In Figure 5, in a short time, the latter is the case, i.e., a purely viscous load with semi-infinite viscous media, which is indeed appropriate for a deposit-free surface in contact with the solvent. However, at 200 s, when deposits on the order of 0.2 μm were observed by SEM imaging, pure viscous scaling was not valid anymore; moreover, the dissipation still kept increasing. This reflects the fact that there is a viscoelastic film on the surface and, as a result, it requires a more complex model, i.e., the model proposed in the Introduction section. Based on these results, the *n* = 3 harmonic was primarily used for analysis.

### Flat Film Approximation

**Figure 6:**
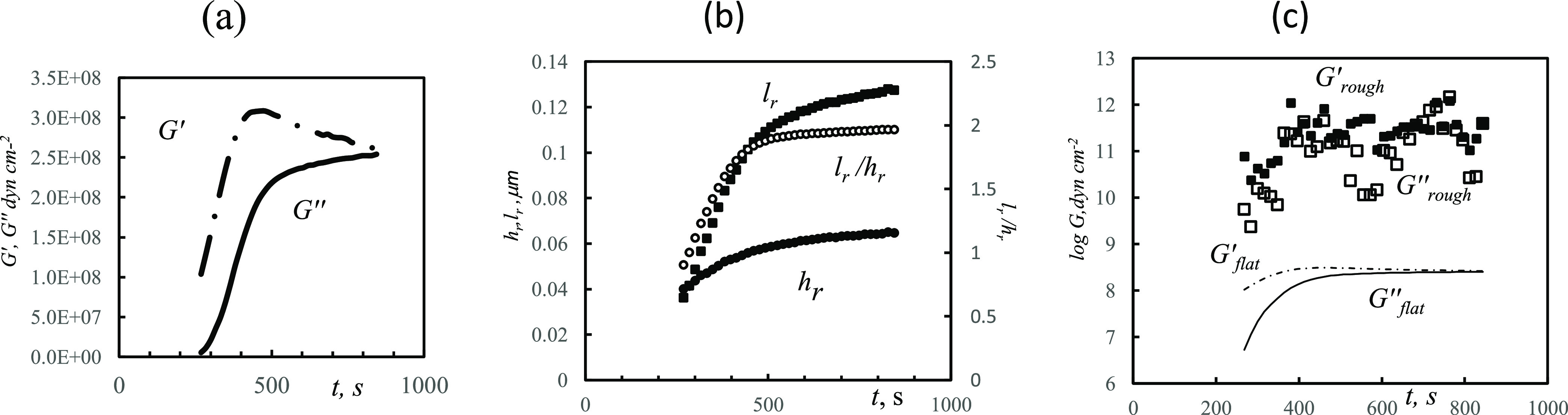
(a) Storage (*G*′-·-) and loss (*G*″ -) moduli at 15 MHz calculated as a function of time (eq 3) for a flat interface using eq 2 for *Z*_L_ for a siderite film precipitating on Au-coated QCMD from 15 mM FeCl_2_, pH 6.8 (with NaHCO_3_), *T* = 80 °C. (b) Parameters (eq 4) of the uneven bumpiness as a function of time calculated employing eq 5 for *Z*_L_^*^. Complex *Z*_L_ is found from eq 3 at the condition that its complex roots remain constant at 400 s < *t* < 850 s and approximately equal to *G*′_∞_ = 4.02 × 10^11^, *G*″_∞_ = 3.86 × 10^11^ dyn cm^–2^. (c) Logarithms of storage *G*′ and loss *G″* moduli for parameters of roughness.

As previously noted, the complex shear modulus, *G*, comprises two components *G*′ and *G*″. Using the Sauerbrey equation, as a first approximation for the thickness, these values can be determined as a function of time. Viscosity of water was used for the liquid (0.0034 g cm^–1^s^–1^ at 80 °C). In the first instance, the film is presumed to be flat, i.e., the use of *Z*_L_ as presented in eq 2 is used in the full eq 3 to calculate *G*. Results for the flat case (Figure 6a) show that until 200 s, the deposited film was too thin and/or inconsistent, so the complex roots cannot be found. This can be attributed to the fact that the deposition of the crystals constitutes individual events; subsequently, they can undergo a variety of possible growth mechanisms, or not, on the surface and form a film. If the crystals’ critical size, *r*_*c*_, is comparable to the film thickness, *h*_*f*_, then the deposit cannot be considered a flat layer; if this is the case, i.e., *r*_*c*_ ≈ *h*_*f*_, then the use of eq 3 is invalid as the necessary flatness criteria cannot be met due to the derivation approach employing propagation of plane waves. This condition corresponds to *r_c_* ∼ 0.2 μm according to Figure 3a, where the thickness of the layer can be estimated from the detached edge. A typical value of *G* ∼ 2 × 10^10^ dyn cm^–2^ from this case study then corresponds to the deformation wavelength in the film . In turn, this makes the exponents in eq 3 ca. 3 when *h*_*f*_ = 0.3 μm. In summary, the closeness of *r_c_* and *h_f_* makes it such that eq 3 cannot be simplified; rather, the exact form is required. The results of calculations assuming a flat film at the liquid interface (Figure 6a) are significantly lower than those reported in the literature^9^ (the storage shear modulus, *G*′, for siderite is 51 GPa (51 × 10^10^ dyn cm^–2^) at room temperature).

### Uneven (Bumpy) Film Approximation

The impedance is transformed to account for surface roughness using a “bumpiness” impedance term, employed in eq 5 or 6; this results in a significant impact on the elastic moduli calculated, as well as on the overall behavior. However, practical implementation is complicated by the fact that there are four unknown parameters (*h*_r_, *l*_r_, *G*′, *G″*) and two equations, one each for the real and imaginary parts in eq 7. Therefore, some actual experiment-related assumptions are inevitable. Even if geometrical roughness parameters are accessible from SEM imaging (or other techniques), unless it is performed in situ during deposition, the final image is the only reliable source for analysis. As such, estimating the time course of roughness evolution can be achieved from QCMD data and eqs 3–7. Herein, we propose a methodology to determine, in a unique way, all parameters by using only one final SEM image.

Inspection of Figures 4 and 5 shows different time scales for frequency and dissipation development. Namely, the frequency decreases significantly (by 24 kHz) until 400 s and then stops. Dissipation increases before and after 400 s albeit at different, slowing, rates. This leads to the conclusion that the bulk of the film is formed in the first stage, by 400 s; this is followed by the second stage during which the thickness of the film does not change but rather the degree of unevenness and/or bumpiness evolves. The second stage is particularly notable as this involves both the roughness amplitude, *h*_r_, and the correlation length, *l*_r_, (eq 4). The geometrical parameters (*l*_r_, *h*_r_) of the final film (*t* = 850 s) can be determined from the SEM image. These results can be substituted into eqs 5 or 6, as appropriate, to give *Z*_L_^*^. In turn, this value can then be used in eq 7, with the pre-existing information that *G* is a mechanical modulus of the flat layer, to give the complex roots of the final film, *G*′_∞_ and *G*″_∞_, similar to the methodology used for flat surfaces. With *G*_∞_ thus established from SEM, one can calculate “backwards in time”: for each earlier time (*t* < 850 s) until just before the inflection point (400 s). Solving eq 3 for the given QCMD data is accomplished by varying *Z*_L_ until *G*′ and *G*″ are the same as those established for the final point *G*′_∞_ and *G*″_∞_ as these values should be the same within the window of *t* = 400–850 s. With the determined complex *Z*_L_, now *h*_r_ and *l*_r_ are calculated, again employing eq 5 or 6. Since *Z*_L_ = Re *Z*_L_ + *i*·Im *Z*_L_, there are two independent equations that have a unique solution for *h*_r_ and *l*_r_ at any moment. This procedure produces a unique set of *h*_r_, *l*_r_, *G*′_∞_, and *G*″_∞_ for the second “roughening” stage and consequently can be used to show the time dependence of the “bumpiness” parameters. This methodology has been implemented on the data set (Figures 4 and 5) to demonstrate time dependence of *h*_r_ and *l*_r_ (Figure 6b).

Time dependence of *h*_r_ and *l*_r_ starts at 400 s; at shorter times, *G* is time-dependent. The maximal elastic moduli found by variation of *h*_r_ and *l*_r_ at the final time point (*t* = 850 s) turned out to be *G*′_∞_ = 4.02 × 10^11^ and *G*″_∞_ = 3.86 × 10^11^ dyn cm^–2^. They are significantly larger than the 2.5 × 10^8^ dyn cm^–2^ calculated (Figure 6a) for a flat interface. One can observe (Figure 6b) that roughness indeed increases with time, which accounts for the slow dissipation increase (Figure 5). This may be interpreted as deposition of rare larger crystals at the final stage of crystallization, which do not form a condensed layer. Practically, the calculations required finding *h*_r_ and *l*_r_ at each *t*, which (1) both gave positive roots *G*′ and *G″*, and (2) both *G*′ and *G″* should be close to *G*′_∞_ = 4.02 × 10^11^ and *G*″_∞_ = 3.86 × 10^11^ dyn cm^–2^. (This resulted in some unavoidable variation of the roots *G*′ and *G″*.) The logarithmic values of the calculated *G*′ and *G″* (Figure 6c) indicate that *G*′ is within an order of magnitude in agreement with literature data and recent DFT calculations.^9,10^ At *t* < 400 s, *G*′ and *G″* values begin to decrease, which corresponds to buildup of the dense film.

### Fe-Coated QCMD

**Figure 7:**
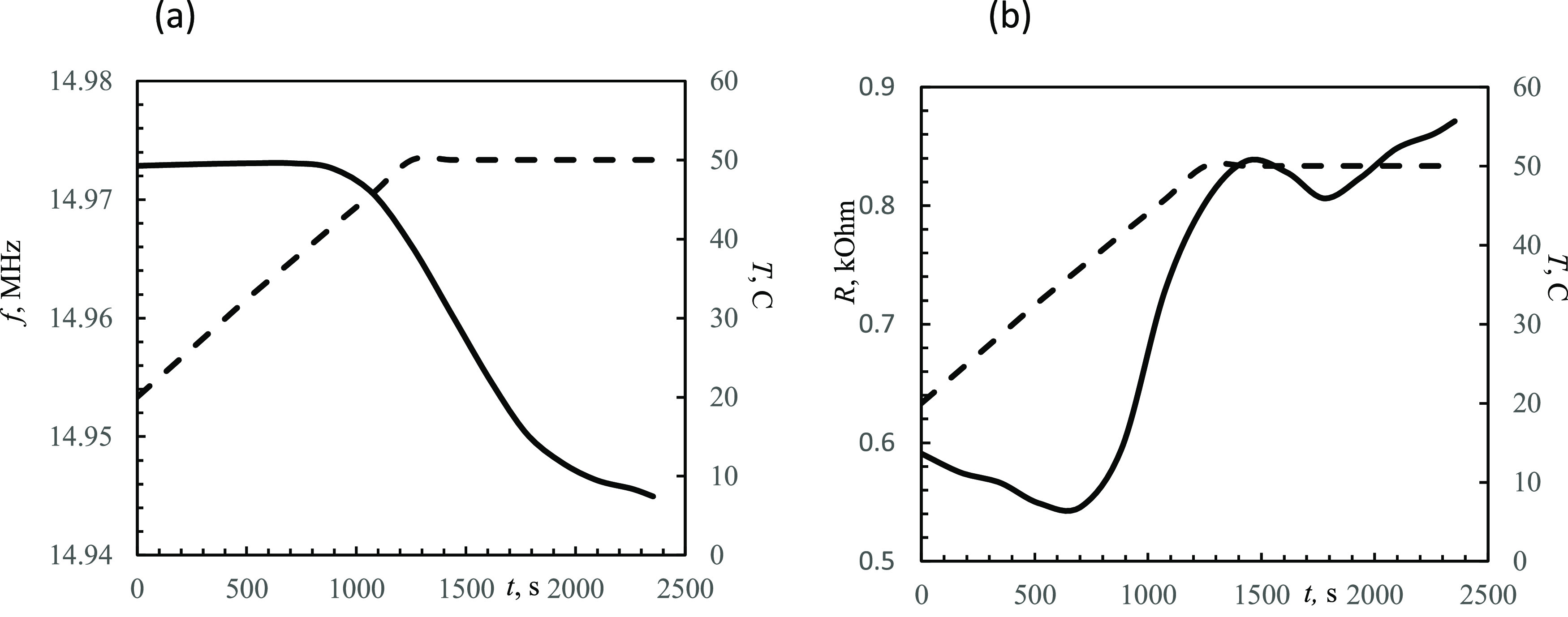
Third resonance frequency (a) and acoustic resistance (b) in electrical units change during siderite precipitation on Fe-coated QCMD. The temperature (- - -) was linearly changed by using Biolin QSense equipment from 20 to 50 °C in 20 min. Accuracy was 0.1 °C. 12.5 mM FeCl_2_, pH 6.8, adjusted with NaHCO_3_.

Crystallization experiments of Fe-coated QCMD were performed with a temperature ramp from 20 to 50 °C over a period of 20 min (temperature gradient of 1.5 °C min^–1^). Data for 15 MHz for both the resonance frequency and acoustic resistance indicate that there is a period with virtually no deposition (Figure 7). At ca. 45 °C, deposition appears to commence. At this temperature, the resistance increases up to 0.3 kΩ, and the frequency drops by 30 kHz. Using the approach described previously, the thickness of the siderite layer can be estimated; in this case study, it is ca. 0.4 μm.

**Figure 8:**
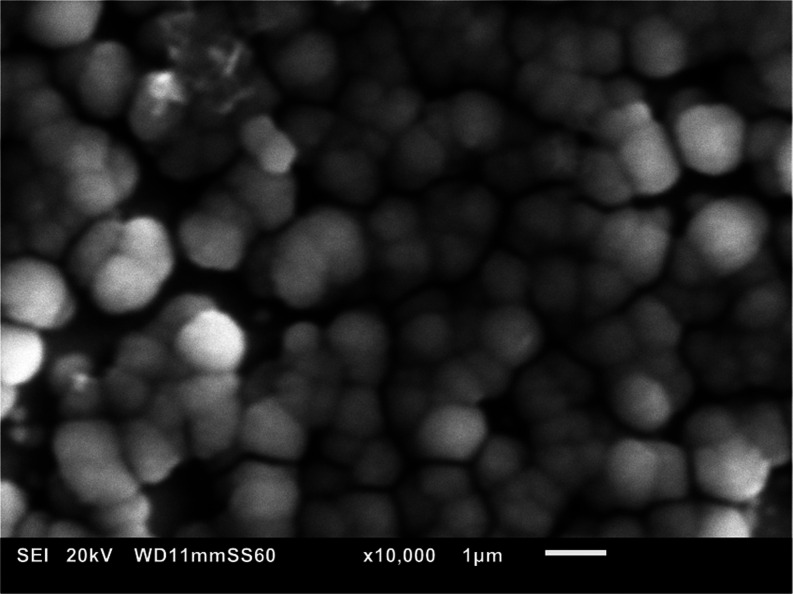
SEM image of the siderite precipitate on Fe-coated QCMD from the experiment in Figure 7.

**Figure 9:**
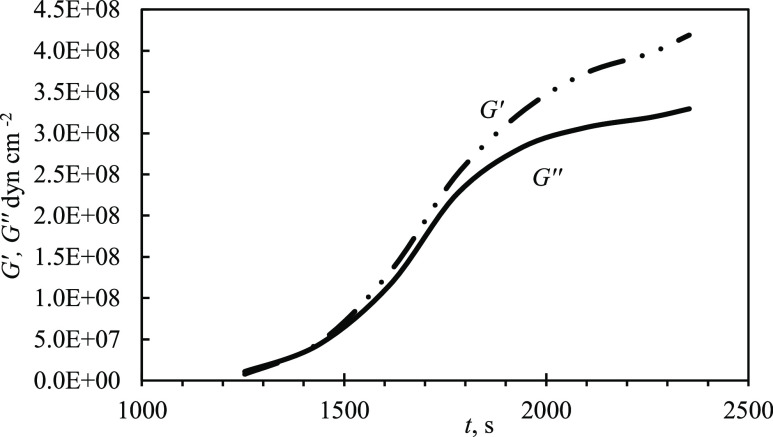
Complex mechanical moduli as a function of time for the experiment depicted in Figures 7 and 8. Third harmonics (15 MHz). *G″* (solid line) and *G*′ (dashed double dotted line) were calculated presuming a flat interface, and therefore employing eq 2 and eq 3.

Images obtained by SEM of the deposit (Figure 8) show a similar structure to deposition on the Au QCMD surface. In this case, calculations of elastic modulus were carried out with viscosity of water 0.0054 g cm^–1^ s^–1^ at 50 °C (Figure 9), presuming a flat interface, and therefore employing eqs 2 and 3.

**Figure 10:**
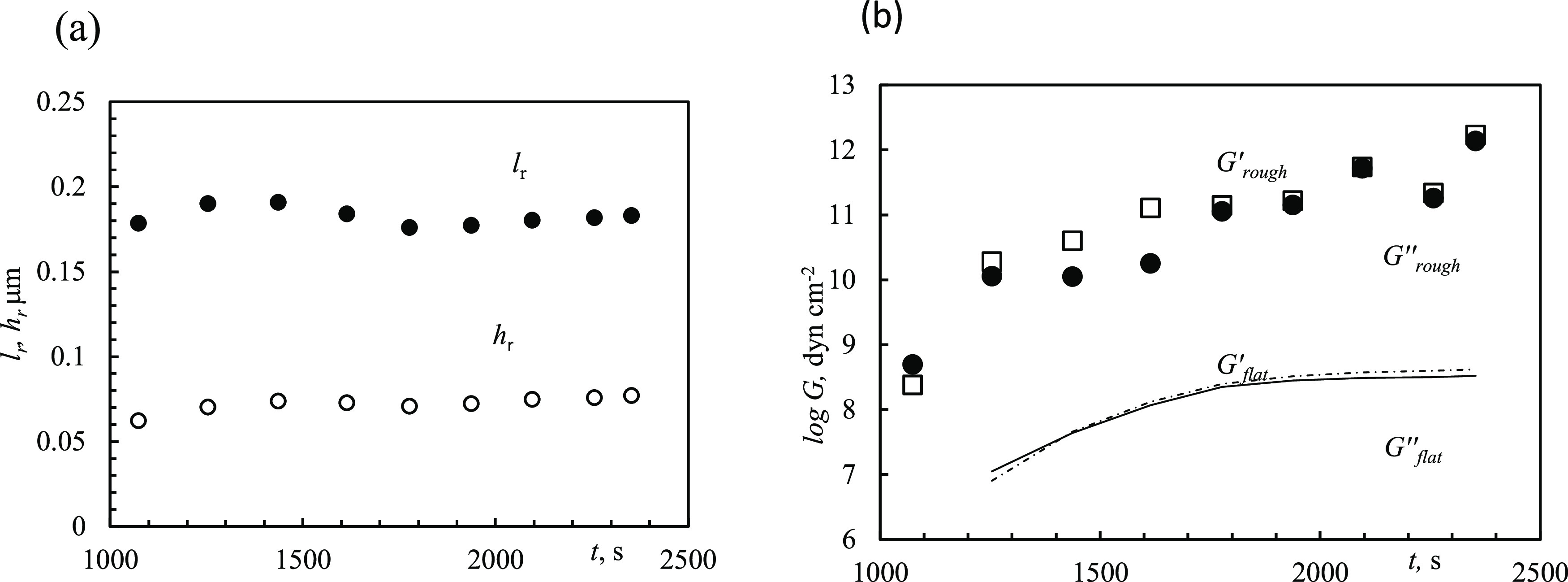
Roughness parameters *l*_r_ and *h*_r_ (a) and corresponding storage and loss moduli (b) as a function of time for FeCO_3_ on Fe QCMD in Figure 7. Values were calculated employing *Z*_L_^*^ from eq 5 in 7 by maximizing positive Re*G* (*G*′) and Im*G* (*G*″). Parameter δ = 0.107 μm.

Unsurprisingly, the estimated values of *G* are low when a flat-interface assumption is used (Figure 10), i.e., *Z*_L_^*^ from eq 5 is used in eq 7. Further, both *l*_r_ and *h*_r_ are estimated according to eq 5 with δ = 0.11 μm (for water at 50 °C). Taking the appropriate roughness terms into account increases both *G*′ and *G*″ by several orders of magnitude and within tens of GPa of the literature value (Figure 10b). Comparing the results on the Au versus Fe-coated sensor shows particles of approximately the same size.

## Discussion

### Mean-Field Calculation of *G* Moduli of a Composite

Elastic storage (*G*′) and loss (*G*″) moduli can be reasonably calculated from acoustic impedance data for siderite precipitates with an appropriate roughness expression; the storage modulus is the same order of magnitude as literature values. There is no data for the loss modulus *G*″ for siderite. One of the reasons may be that QCMD measures losses in a composite layer, consisting of spherical particles of siderite separated by a water layer. A mean-field theory, analogous to the Clausius–Mossotti approximation for effective elastic moduli μ_eff_, was developed.^11^ Here, the first approximation is used, which can be represented in the closed form.
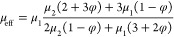
8where μ_eff_, μ_1_, and μ_2_ are complex shear moduli of a composite comprising phase 1 and phase 2. Phase 1 is an incompressible solvent separating hard spheres of phase 2, siderite in this case; there is a volume fraction, ϕ, in the composite. From SEM imaging, the layer indicates a quite dense packing, suggesting that it is close to 1. It is essential that the solid matrix comprises of spheres, but diameters can be arbitrary. Experimentally, the value of the effective moduli of the composite is measured, μ_eff_ = *G*′ + *i*·*G*″. For simplicity, one can assume that the siderite phase is an ideal solid; therefore, μ_2_ only has a real part: μ_2_ = *G*′_0_; for the liquid, μ_1_, one can state that μ_1_ = *i*ωη. Let us assume that all losses are happening in the liquid between spheres. In eq 8, after the separation of real and imaginary parts, a simpler form (eqs eq 8a and eq 8b) can be used to analyze the experimentally accessed *G*′ and *G″*.
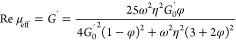
8a
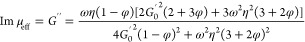
8bThe losses appear due to the presence of a solvent between ideal solid-phase particles. It must be considered that, as with any Clausius–Mossotti type of equation, accuracy is at its peak for ϕ ∼ 0.5 and fails as ϕ → 1; the latter occurs as the mean-field approximation is not enough. However, eq 8a and eq 8b have a formally correct limit at ϕ = 1 (no solvent): μ_eff_ = *G*′_0_, while being a smooth function of ϕ. Therefore, one can attempt to estimate the influence of a solvent at ϕ → 1. The experimental result *G*′ ∼ *G*″ ∼ *G*′_0_ requires ωη ∼ (1 – ϕ)*G*′_0_. For a 1% solvent content (1 – ϕ ∼ 0.01), with *G*′_0_ ∼ 10^11^ dyn cm^–2^ and ω ∼ 2π × 15 × 10^6^ Hz, one can estimate the viscosity of a solvent inside the matrix η ∼ 10 g cm^–1^ s^–1^. This value exceeds the viscosity of water. This can be understood in terms of the Brinkmans equation^12,13^ for porous media, where an effective viscosity coefficient and porous media resistance are introduced. Calculations of the former^14^ show that it can exceed the normal viscosity coefficient by hundreds of times in the limit ϕ → 1.

### Kinetics of Precipitation

Following precipitation, ripening and coalescence phenomena begin to influence the evolution of the particle size distribution. These phenomena may become dominant once the driving force for precipitation and growth is exhausted. The theory is given in terms of change in the size distribution function in the crystal size space, governed by the continuity equation.^15^ Smaller particles appearing during nucleation dissolve and produce larger particles. The resulting equations indicate that the average size remains constant, while the number of crystals *N* decreases with time *t* as
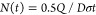
9where *Q* is the initial oversaturation and *D* is the diffusion coefficient. The parameter , where α is the surface tension on the siderite–water interface, *v*′ is the molecular volume of dissolved iron carbonate, and *c*_0∞_ is the equilibrium concentration (volume/volume) above the flat siderite–water interface. The average size of particles, *a*, is equal to the critical size and increases over time as
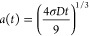
10

The surface of QCMD serves as a sink for crystals, and it also, in a certain sense, “memorizes” the critical size progress as a function of time because lower layers are formed earlier in time than the upper layers. This is a consequence of the fact that the size distribution function has a sharp peak around the critical size. According to eqs 9 and 10, lower layers should have more crystals of a smaller diameter than the upper layers. From eqs 9 and 10, it also follows that the product *N*(*t*)*a*(*t*)^3^ is independent of time. Figure 5 shows an illustrative and representative image depicting five crystals of ca. 4 μm diameter situated on top of a layer containing ca. 300–400 crystals of ca. <0.5 μm diameter, where it is evident that the crystals found in the underlying layer were deposited before the ones positioned on top. The product *Na*^3^ calculated for the upper layer 5 × 4^3^ = 320 matches approximately the product 350 × 1^3^ for the lower one. This conclusion seems straightforward, but it is far from trivial, for as stated before, it follows from the fact that the size distribution function sharply peaks around the critical size; therefore, one can follow only its development with time.

## Conclusions

The formation of films, comprising deposited siderite particles, on Au- and Fe-coated quartz was monitored by QCMD. In all of the cases, XRD analysis showed the particles to be pure siderite. The change in resonance frequency at 15 MHz as well as dissipation was interpreted as the growth of a siderite film with increasing unevenness (bumpiness). Mechanical shear moduli were calculated using established physical chemistry models, taking roughness and the presence of a trapped solvent into consideration. The structure of the deposit with a dense initial layer of small crystals and a disordered upper layer of larger crystals agrees with nucleation followed by a coalescence process. These measurements provide information about the evolution of the deposited film, including thickness and mechanical properties. This approach can be used to assess the extent to which different types of films, growing under different conditions, can promote or inhibit corrosion or, more broadly, be applied to assess deposition and formation. Overall, the workflow presented here provides a paradigm on how the QCMD can be used, in tandem with established physical chemistry theories and complementary experimental techniques, to facilitate the analysis of complex films, comprising individual deposited particles formed upon reaction in a bulk liquid.
